# First-Time Identification of the PPAT Protein as a Novel Antibacterial Target: Antimicrobial and Antioxidant Insights from E. purpurea

**DOI:** 10.1021/acsomega.5c00273

**Published:** 2025-05-21

**Authors:** Safiye Elif Korcan, Nevin Çankaya, İbrahim Bulduk, Serap Yalçin Azarkan, Şah İsmail Çivi

**Affiliations:** † 175652 Usak University, Vocational School of Health Services, Usak 64200, Turkey; ‡ Afyon Kocatepe University, Faculty of Engineering, Department of Chemical Engineering, Afyonkarahisar 03200, Turkey; § Kırsehir Ahi Evran University, Faculty of Medicine, Department of Medical Pharmacology, Kırşehir 40100, Turkey; ∥ Usak University, Faculty of Engineering, Department of Molecular Biology and Genetics, Usak 64200, Turkey

## Abstract

Echinacea purpurea (Asteraceae) is a perennial medicinal herb with immune-stimulating and anti-inflammatory properties. In this study, the antioxidant and antibacterial properties of the organs of the E. purpurea plant were investigated, and significant amounts of total phenolic and total flavonoid contents were detected in flower extracts. It was determined that the DPPH% values of the water extract were higher than the values of methanol extracts. It was observed that Fe^3+^ reduction capacity increased as the concentration increased in all plant extracts. The highest antimicrobial activity was determined to be against Pseudomonas aeruginosa (35 mm) and Klebsiella pneumonia (20 mm) at the petal (*Ep*P)–methanol extract. In HPLC analysis of component-based phenolic substances, protocatechuic acid, caffeic acid, coumaric acid, chlorogenic acid, and ferulic acid were determined in the extracts. The lowest protein–ligand binding energy (kcal/mol) was found to be −9.6 kcal/mol in chlorogenic acid. Chlorogenic acid can bind with PPAT’s T10­(B)­OG1, W12­(b), I127­(B)­O, and S129­(B)­OG and P88­(B)­HN2, T10­(B)­N, F11­(b)­N, D12­(B)­N, S129­(B)­N, and K12­(B)­NZ amino acid residues via hydrogen bonds. The evidence collected indicates that chlorogenic acid inhibits phosphopantetheine adenylyltransferase (PPAT), validating PPAT as a potential target for antibacterial therapy for the first time. The development of selective inhibitors such as chlorogenic acid of bacterial PPAT is promising for the discovery of new antibiotics.

## Graphical abstract

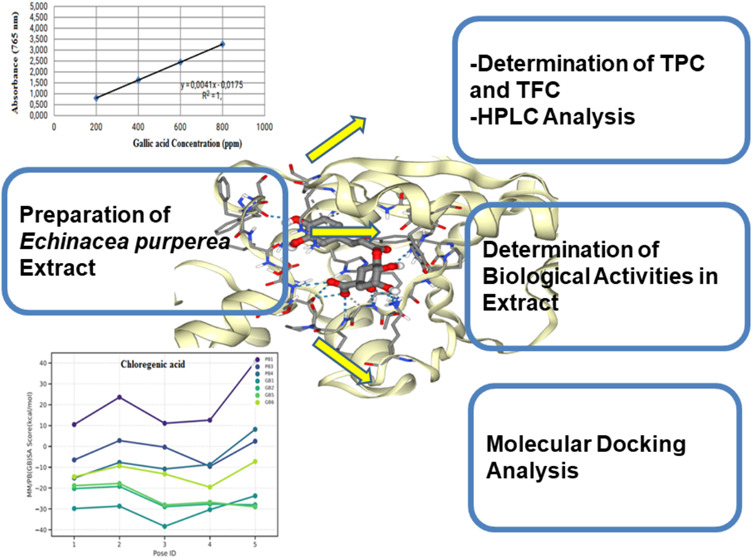

## 1 Introduction

Most of the antibiotics in clinical use today still belong to structural classes discovered in the middle of the last century, and the steady spread of resistance to these antibiotics poses a significant threat to the treatment of infectious diseases worldwide. With the emergence of multidrug-resistant (MDR) and pan-drug-resistant (PDR) bacteria, the World Health Organization (WHO) and the Centers for Disease Control and Prevention (CDC) warn of the “postantibiotic era”.^1^ The strategy of discovering new antibacterial drugs by modifying antibiotic structures is a valid strategy today, but the development of compounds targeting new cellular targets has become imperative today. For example, recently, activation of β- and γ-carbonic anhydrases with tripeptides in pathogenic bacteria and new teixobactin analogues containing a total lactam ring have been important in antibacterial treatment research. β- and γ-CA isoenzymes found in bacteria play critical roles in pH balance, ion transport, and metabolic processes. Teixobactin is a new antibiotic discovered in 2015 and is particularly effective against Gram-positive bacteria. It exhibits bactericidal activity by binding to lipid II and lipid III, which are critical for cell wall synthesis. It is particularly effective against pathogens such as methicillin-resistant Staphylococcus aureus (MRSA) and Streptococcus pneumoniae. The lactam ring in the structure of teixobactin plays an important role in its antibacterial activity. Therefore, synthesis and testing of new teixobactin analogues containing the lactam ring may provide more effective treatment options against resistant bacteria.^2,3^

Additionally, pantetheine-phosphate adenylyltransferase (PPAT, also known as CoaD) is a novel target that has recently attracted attention for its antimicrobial activity. The hexameric enzyme PPAT catalyzes the penultimate step in coenzyme A (CoA) biosynthesis.^1^ Although the CoA biosynthesis pathway is similar in all life forms, the sequence similarity between prokaryotic and eukaryotic enzymes is very low.^4^ Therefore, it may be possible to selectively target enzymes in the CoA biosynthesis pathway using bacterial PPAT inhibitors^5^ and contribute to the creation of a new effective antimicrobial class. Today, sulfonamides are used as antibacterial agents that inhibit dihydropteroate synthetase, which is involved in the biosynthesis of the cofactor folate.^6^

Phenolic compounds and flavonoids carrying a hydroxyl group in their aromatic ring have long been used as antimicrobials, anti-inflammatories, immune system enhancers, and antioxidants.^7^ Research on these phytochemicals obtained from various medicinal plants is still of interest. Echinacea purpurea (E. purpurea) has been used as a medicinal plant since the beginning of modern medicine, and therefore, it is and will continue to be a valuable source of molecules for drugs.^8^ E. purpurea is a perennial herbaceous plant native to North America and belongs to the Asteraceae family.^9,10^ Traditionally, it has been utilized in herbal medicine to address various ailments, particularly those related to the respiratory system.

E. purpurea is renowned for its potential health benefits, primarily attributed to its rich phytochemical composition. The plant contains alkamides, caffeic acid derivatives, polysaccharides, glycoproteins, and flavonoids, such as nicotiflorin and rutin. Chicoric acid is the predominant phenolic acid in E. purpurea. Studies have reported its concentration in roots ranging from 1.68 to 2.27%, and in aerial parts from 0.52 to 2.02%, depending on the season. Caftaric acid, a significant phenolic compound, has been identified in the roots and aerial parts of *E. purpurea*. Its content varies between 0.18 and 0.82%, influenced by seasonal changes.^11^

Kaempferol derivatives are among the primary flavonoids in E. purpurea. Compounds such as kaempferol-*O*-deoxyhexosyl-hexoside and kaempferol-3-*O*-rutinoside have been identified. Flavonoids such as quercetin-*O*-deoxyhexosyl-hexoside and quercetin-*O*-malonylhexoside have been detected in E. purpurea extracts. These compounds are known for their antioxidant properties.^12^ Research has identified specific compounds in E. purpurea, such as purpureaterpene E, that exhibit significant anti-inflammatory activity. Purpureaterpene E has been shown to inhibit nitric oxide production in LPS-induced macrophages, potentially via the NF-κB signaling pathway.^13^ Flavonoids and phenolic compounds are the most important components of E. purpurea (L.) Moench. However, the concentration of chicoric acid, a caffeic acid derivative, varies according to both E. purpurea species and organ type, as well as cultivation conditions and environmental factors.^8^

Despite promising findings, several research gaps remain. First, the variability in chemical composition of E. purpurea extracts due to differences in extraction methods, plant parts used, and cultivation conditions poses challenges in terms of consistency and reproducibility in studies, and although some compounds have demonstrated in vitro bioactivity, docking studies are needed to elucidate the underlying mechanisms and confirm the therapeutic potential. Therefore, this study aimed to identify phenolic compounds with significant biological activity in E. purpurea water and ethanol extracts obtained by brewing and ultrasonic extraction and to explain their mechanisms of action through molecular docking.

## 2 Materials and Methods

Folin–Ciocalteu reagent (2 M), gallic acid (≥99.0%), catechin (≥99.0%), aluminum chloride hexahydrate (≥99.0%), methanol (≥99.8%), glacial acetic acid (≥99.7%), acetonitrile (≥99.5%), sodium carbonate (≥99.5%), potassium ferricyanide (≥99.0%), and sodium nitrite (≥99.0%) were supplied by Sigma-Aldrich Co. (Istanbul, Turkey). Ultrapure water with a conductivity of less than 0.05 μS cm^–1^ used in the experimental studies was produced using the Milli-Q System. All other chemicals were of analytical grade.

### 2.1 Preparation of Plant Extracts

E. purpurea plant was obtained from plants grown in the fields of Uşak University Faculty of Agriculture, and species identification was done using The World Flora Online (www.worldfloraonline.org/taxon/wfo-0000036347).

The brewing method is a low-energy method. Depending on the heat and time, it can be effective in preserving heat-sensitive compounds, such as polyphenols and flavonoids. However, it may be difficult to completely release target compounds in plants with hard cell walls. However, it can help to preserve delicate compounds. In ultrasonic extraction, the cavitation created by sound waves breaks down cell walls, and the content is released into the solvent quickly. This method can generally increase the extraction of phenolic compounds and antioxidants. However, high energy input can cause deterioration in heat-sensitive compounds. In this study, both brewing and extraction with ultrasonically sound waves were performed. Water and ethanol were used as solvents in the extraction. Ethanol increases the solubility of polyphenols, flavonoids, and some lipophilic compounds. It usually provides extracts with high antioxidant activity. Water, on the other hand, provides the dissolution of hydrophilic compounds, such as polysaccharides and tannins. It is generally suitable for the extraction of more polar compounds.

After the plant was separated into its organs, such as bud, root, lower leaf, upper leaf, petal, flower, root, and stem, it was dried at 27̊C ± 2 for 15 days in a dark environment. Dried samples were ground and passed through an 80 mesh sieve. 500 mg of dried and ground samples were precisely weighed and 50 mL of solvent (35 mL methanol + 15 mL water) was added, followed by extraction in an ultrasonic bath for 30 min. Extracted samples were filtered through white band filter paper and transferred to Falcon tubes for analysis. For extraction with water, 500 mg of the sample was again precisely weighed, and 50 mL of boiled water was added and extracted by the brewing method. The extracted samples were filtered through filter paper and transferred to coded Falcon tubes (Table 1). The extracts were stored in a refrigerator (+4 °C) until analysis.

**Table 1:** DPPH (%), Optical Density, and Values of TFC and TPC in Methanol and Water Extracts of E. purpurea

	TPC (GAE/g)		TFC (CAE/g)		DPPH (%) of E. purpurea extracts	
	methanol (M)	water (W)	methanol (M)	water (W)	methanol (M)	water (W)
lower leaf (*Ep*LL)	0.813 ± 0.108(202.56)^a^	0.678 ± 0.295(170.74)^b^	0.030 ± 0.011(305.00)^c^	0.018 ± 0.013(185.00)^d^	82 ± 0.02^e^	87 ± 0.05^e^
upper leaf (*Ep*UL)	1.034 ± 0.086(256.46)^a^	0.764 ± 0.457(192.69)^b^	0.033 ± 0.011(335.00)^c^	0.022 ± 0.018(225.00)^d^	85 ± 0.03^e^	86 ± 0.02^e^
petal (*Ep*P)	2.085 ± 0.650(512.80)^a^	1.940 ± 0.506(478.05)^b^	0.089 ± 0.010(895.00)^c^	0.059 ± 0.043(595.00)^d^	80 ± 0.01^e^	84 ± 0.06^e^
bud (*Ep*B)	1.008 ± 0.324(250.12)^a^	0.968 ± 0.425(241.47)^b^	0.029 ± 0.020(295.00)^c^	0.027 ± 0.015(275.00)^d^	53 ± 0.02^e^	57 ± 0.05^e^
flower (*Ep*F)	0.569 ± 0.121(143.05)^a^	0.589 ± 0.178(148.78)^b^	0.016 ± 0.010(165.00)^c^	0.018 ± 0.009(185.00)^d^	32 ± 0.10^e^	38 ± 0.05^e^
root (*Ep*R)	0.283 ± 0.027(74.75)^a^	0.283 ± 0.027(73.17)^b^	0.010 ± 0.006(100.00)^c^	0.011 ± 0.003(115.00)^d^	26 ± 0.02^e^	29 ± 0.08^e^
stem(*Ep*S)	0.560 ± 0.138(140.85)^a^	0.387 ± 0.108(100.00)^b^	0.016 ± 0.009(105.00)^c^	0.014 ± 0.006(145.00)^d^	86 ± 0.01^e^	89 ± 0.04^e^

a Significant difference in the TPCM of the extract (*P* < .05).

b Significant difference in the TPCW of the extract (*P* < .05).

c Significant difference in the TFCM of the extract (*P* < .05)

d Significant difference in the TFCW of the extract (*P* < .05).

e Positive correlation between TPC and TFC amounts and DPPH (%).

### 2.2 Determination of Flavonoids and Phenolic Compounds by Biochemical Analysis

#### 2.2.1 Determination of Total Phenolic Content (TPC) and of Total Flavonoid Content (TFC)

The total phenolic content (TPC) of plant extracts (500 mg/50 mL) was determined using the Folin–Ciocalteu method.^14^ In brief, 500 μL of water and methanol extract were pipetted into a glass tube, 250 μL of Folin reagent and 7250 μL of deionized water were added, and the mixture was kept in the dark for 5 min. Then, 2000 μL of 7.5% sodium carbonate (Na_2_CO_3_) was added and kept in the dark for another 30 min. The absorbance value was measured using a UV spectrophotometer (Shimadzu UV-1800) at 760 nm, and the amount of TPC was calculated by using the gallic acid standard curve. The results were expressed as gallic acid equivalents (GAE) per gram.

Total flavonoid content (TFC) was measured using the aluminum chloride (AlCl_3_) colorimetric method.^15^ Briefly, 50 μL of the extract solution (500 mg/50 mL) was pipetted into a glass tube, followed by 950 μL of methanol and 6400 μL of deionized water. Then, 300 μL of 5% sodium nitrite (NaNO_2_) and 300 μL of 10% AlCl_3_ were added, and the mixture was allowed to react for 5 min. Then, 2000 μL of 4% sodium hydroxide (NaOH) was added, and the mixture was kept in the dark for 15 min. Absorbance values were measured using a UV spectrophotometer (Shimadzu UV-1800) at 510 nm, and TFC values were calculated using a catechin standard curve; the results were expressed as catechin equivalents per gram (CAE).

#### 2.2.2 HPLC Analysis

Standards for HPLC analysis, including gallic acid, protocatechuic acid, chlorogenic acid, vanillic acid, syringic acid, caffeic acid, coumaric acid, ferulic acid, and sinapic acid, were sourced from Sigma-Aldrich. The phenolic acid content in the samples was analyzed using an Agilent 1260 HPLC system, which featured a UV detector, a quadruple gradient pump, automatic sampling, and Chemstation software. Separation was carried out on an ACE-C18 column (4.6 × 150 mm, 5 μm).

Ultrapure water (A) and acetonitrile (B) containing 0.1% acetic acid were used as the mobile phases. The flow rate was maintained at 1.0 mL/min, with the column temperature set at 25 °C and an injection volume of 10 μL. The detection wavelengths were selected based on the maximum absorption of the phenolic compounds: Caffeic acid and chlorogenic acid were detected at 330 nm, coumaric acid at 305 nm, syringic acid, protocatechuic acid, and gallic acid at 280 nm, and vanillic acid at 225 nm.^16^

### 2.3 Determination of Biological Activities in Extracts

#### 2.3.1 DPPH (2,2′-Diphenyl-1-picrylhydrazyl radical) Assay

The antioxidant activity of the extracts was assessed using the DPPH radical scavenging method.^17^ To prepare the DPPH stock solution, 0.0024 g of DPPH was accurately weighed and dissolved in 100 mL of methanol, resulting in a concentration of 6 × 10^–5^ M. A working solution with a concentration of 40 mg/L was then prepared by diluting the stock solution with methanol. For the assay, 300 μL of the extract was mixed with 5700 μL of the DPPH working solution in a 10 mL test tube. The mixture was incubated in the dark at room temperature for 60 min. After incubation, the absorbance was measured at 517 nm using a Shimadzu UV-1800 spectrophotometer with ultrapure water as the blank. A control solution (without the extract) was also prepared, and its absorbance was measured at the same wavelength. The antioxidant activity was calculated using the following formula

$$a\mathrm{ntioxidant activity}\left(\%\right)=\left[\left(\mathrm{AC}{\left(0\right)}_{517}-\mathrm{AA}{\left(t\right)}_{517}\right)/\mathrm{AC}{\left(0\right)}_{517}\right]\times 100$$

where AC(0)_517_ is the absorbance of the control at *t* = 0 min and AA­(*t*)_517_ is the absorbance of the antioxidant at *t* = 1 h.

#### 2.3.2 Fe^3+^ Reduction Capacity Determination

The Fe^3+^ reduction capacity was measured following the method of Oyaizu.^18^ In this process, 2.5 mL of phosphate buffer (0.2 M, pH 6.6) and 2.5 mL of 1% potassium ferricyanide (K_3_Fe­(CN)_6_) were combined with different volumes of the sample (100, 200, 300, 400 μL) and incubated at 50 °C for 20 min. Afterward, 2.5 mL of 10% trichloroacetic acid (TCA) was added, and the mixture was centrifuged at 2500 rpm for 10 min. After centrifugation, 2.5 mL of the supernatant was collected and brought to a final volume of 5 mL by adding 0.5 mL of a 0.1% ferric chloride (FeCl_3_) solution. The absorbance was then measured at 700 nm by using a spectrophotometer to determine the Fe^3+^ reduction capacity.

The percent inhibition of ferrozine Fe^2+^ complex formation was calculated using the following equation

metal chelating capacity(%)=[(Acontrol−Asample)/Acontrol]×100

#### 2.3.3 Determination of Potent Antimicrobial Activity by the Disk Diffusion Method

The disk diffusion method is frequently used to determine the antibacterial activity of natural products containing complex mixtures, such as herbal extracts. With this method, the diffusibility and effect diameter of the compounds are evaluated, and preliminary information about the effectiveness is obtained. Since the aim of our study is to evaluate the general antibacterial effect of the E. purpurea methanol extract, the disk diffusion method was preferred. MIC determination may be more appropriate when the detailed dose–response relationship is examined on isolated compounds. In this method, the diameter of the inhibition zones created in the Petri dish is measured to determine the resistance or sensitivity of the microorganism to a substance. In the study, Gram-positive Pseudomonas aeruginosa ATCC 11778, Klebsiella pneumoniae NRLLB 4420, Escherichia coli ATCC 35213, and Gram-negative Staphylococcus aureus 25292 microorganisms were used. After the microorganisms were incubated in Nutrient Broth medium at 37 °C until they reached 0.5 McFarland (10^8^ CFU/mL) turbidity, 100 μL was taken and spread homogeneously on the agar surface with sterile swab sticks to cover the entire surface of the Nutrient agar solid medium. Penicillin 10 μg/disc (P10), Chloramphenicol 30 μg/disc C(30), and Erythromycin 15 μg/disc E(15) were used as positive controls.^19^ Discs impregnated with dimethyl sulfoxide (DMSO) dissolved in 20 μL of plant extract were placed on the agar surface to be placed in Petri dishes. After the prepared Petri dishes were kept in the incubator at 37 °C for 24 h, the inhibition zones (zone diameters) around the discs were measured.

### 2.4 In Silico Analysis

The 3D structures of the PPAT protein (ID: 1qjc) were obtained from the RCSB PDB protein database (https://www.rcsb.org/) (Figure 1). The 3D structures of ferulic acid, chlorogenic acid, protocatechuic acid, and vanillic acid were found in the PubChem database (https://pubchem.ncbi.nlm.nih.gov/).

**Figure 1:**
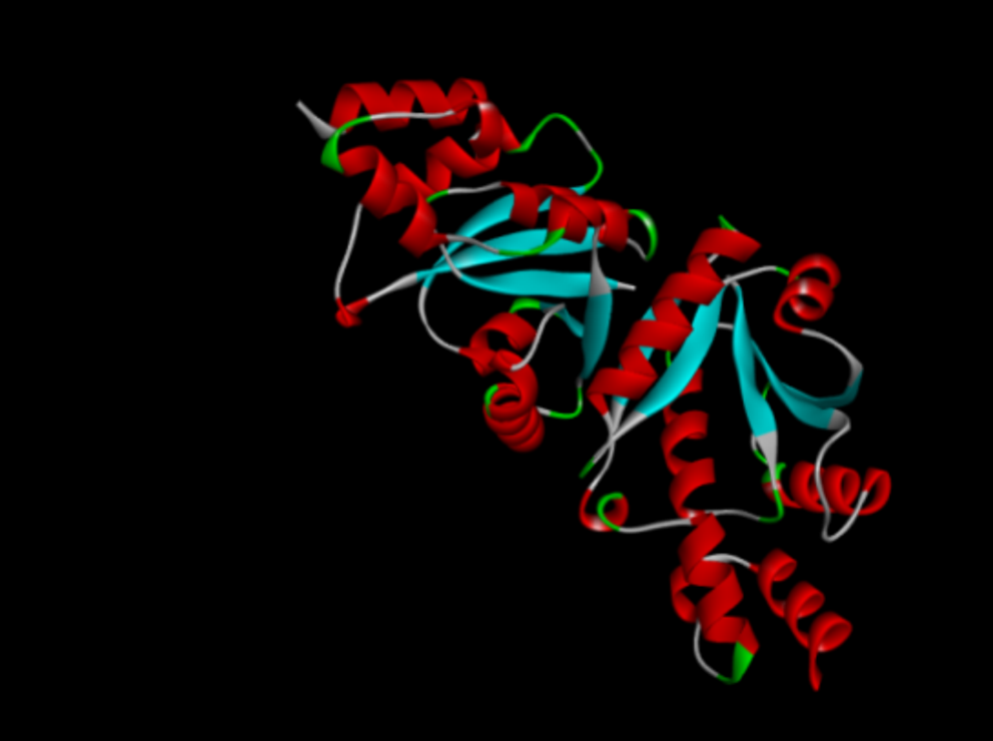
Phosphopantetheine adenylyltransferase (PPAT) (PDB ID: 1qjc).

Molecular docking calculations were validated using both Seamdock and Autodock.^20–23^ To determine the correct binding positions for the ligands, we used the performance of MM/PB­(GB)­SA, including the Schrödinger package and the Amber package (http://cadd.zju.edu.cn/farppi). A dynamic simulation of the ligand and protein complex was performed using the WebGro application.^24–27^ A molecular dynamics simulation was performed for 50 ns to study the stability of the ligand and protein complex.

### 2.5 Determination of the Pharmacokinetics/ADMET Profile

To evaluate the pharmacokinetic properties of ferulic acid, chlorogenic acid, protocatechuic acid, and vanillic acid, ADMET (absorption, distribution, metabolism, excretion, and toxicity) profiles were determined using the Swiss Institute of Bioinformatics online software SwissADME.^28^ (https://www.swissadme.ch/)

### 2.6 Statistical Analyses

The correlation between the TPC and TFC amounts and DPPH (%) was determined by Kendall’s tau_b test in the SPSS 28.0 program. A significant difference in the amount of TPC and TFC and DPPH (%) among the extracts was determined by the Friedman test in the SPSS 28.0 program.^29,30^

## 3 Results and Discussion

### 3.1 TPC, TFC, and HPLC Analysis

E. purpurea is considered an important medicinal plant due to its pharmacological properties such as anti-inflammatory, antioxidant, and immunostimulant.^31^ In this study, TPC results were calculated according to the prepared gallic acid calibration curve (*y* = 0.0041*x* – 0.0175 *R* ^2^ = 1), which is given as (GAE)/g (Table 1). It was determined that the TPC amount of E. purpurea plant extracts was especially high in *Ep*P. It was observed that the methanol extract of *Ep*P had the highest TPC value, 512.80 GAE/g. This was followed by the *Ep*P water extract with 478.05 GAE/g and the *Ep*UL methanol extract with 256.46 GAE/g. The lowest TPC content was determined to be in the water extract of *Ep*R (73.17 GAE/g) and ethanol *Ep*R extracts (74.75 GAE/g). The highest TFC values were found in the *Ep*P methanol extract (895 CAE/g) and water extract (595 CAE/g; Table 1). It was determined that the difference between the TPC and TFC amounts among the extracts was significant (*P* < .05). Sharif et al. found the highest TPC level in water extracts of E. purpurea leaves and flowers, while they also determined high TPC values in methanol flower extracts of the plant. In their study, they reported that leaf extracts contained phenolic levels higher than those of flowers. They detected higher TFC levels in the methanol extracts. The lowest TFC was determined in the methanol extract of flowers.^32^ Various studies showed different phenolic and flavonoid levels in E. purpurea. The reason for this can be explained by factors such as Echinacea species diversity, different geographical conditions, and cultivation conditions.^33^ The aerial parts and roots of E. purpurea are rich in phenols and total flavonoids, and this extract is associated with higher antioxidant activity found in in vitro assays. The improvement of the immune and antioxidant effects of E. purpurea has been associated with polyphenol compounds, such as caftaric acid. Among all caffeic acid derivatives, caftaric acid and chlorogenic acid can act as strong antioxidants against free radicals.

Syringic acid and sinapinic acid were not detected in any of the *Ep* extracts. Ferulic acid (15.61 mg/100 g) was found the most in *Ep*P-M, followed by chlorogenic acid (10.12 mg/100 g). Gallic acid was found in *Ep*LL-M (8.27 mg/100 g) and *Ep*UL-M (3.33 mg/100 g). Protocatechuic acid was detected with a maximum of 8.88 mg/100 g in *Ep*BF-M, and coumaric acid was detected with a maximum of 9.86 mg/100 g in *Ep*R-M. Vanillic acid was only determined at EpP-M (8.76 mg/100 g). Gallic acid and catechin calibration curves are given in Figure 2. HPLC analysis results of the E. purpurea plant are given in Table 2.

**Figure 2:**
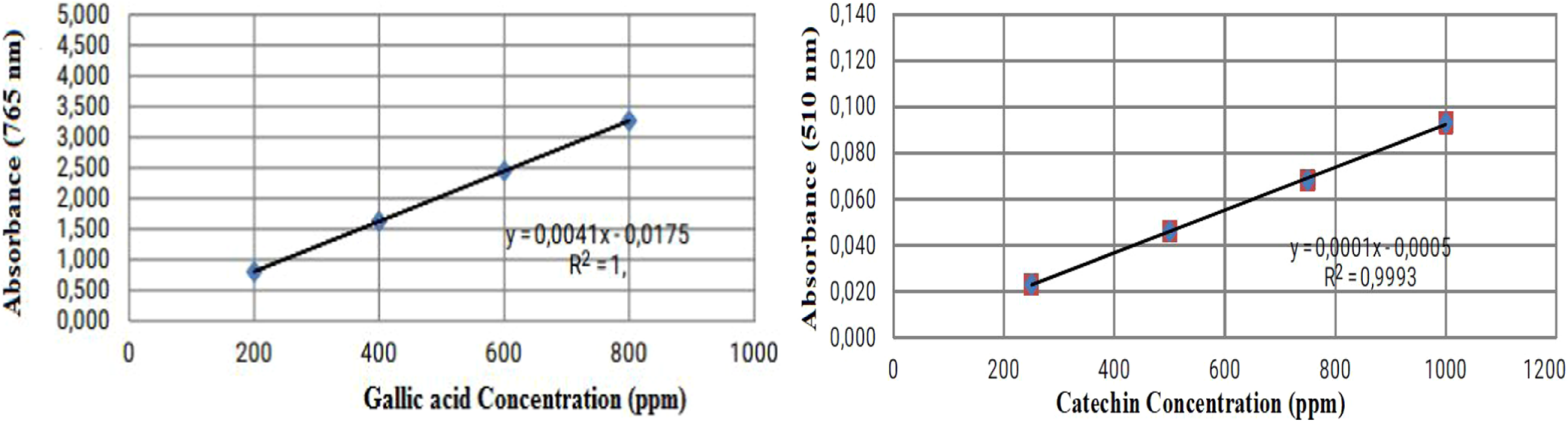
Gallic acid and catechin calibration curves.

**Table 2:** HPLC Analysis of the E. purpurea Plant Material^a^

host plant material HPLC analysis (mg/100 g dry weight)									
extract	gallic acid	protocatechuic acid	chlorogenic acid	vanillic acid	syringic acid	caffeic acid	coumaric acid	ferulic acid	sinapinic acid
*Ep*LL-M	8.27		8.20				8.74	1.14	
*Ep*LL-W		1.05				6.83	7.06		
*Ep*UL-M	3.33		7.88				3.15		
*Ep*UL-W		1.90	8.20						
*Ep*P-M		5.78	10.12	8.76			2.26	15.61	
*Ep*P–W							1.57	7.30	
*Ep*B-M		1.14						3.83	
*Ep*B–W		1.13	4.37						
*Ep*F-M		8.88							
*Ep*F–W		1.11	2.83						
*Ep*R-M							9.86		
*Ep*R-W							3.37	1.07	
*Ep*S-M							8.52	5.35	
*Ep*S–W						7.08	2.09	5.72	

a M: methanol extract, W: water extract.

One of the phytochemical phenolic derivatives, 4-hydroxy-3-methoxycinnamic acid, is known as ferulic acid. In addition to preventing lipid peroxidation by binding transition metals such as iron and copper, ferulic acid has an antioxidant effect as it is an inhibitor of the enzyme that catalyzes the production of free radicals.^34,35^ Ferulic acid is known to have antibacterial and anti-inflammatory, antioxidant, and antitumor effects.^36^ In addition, recent studies have reported that ferulic acid derivatives exhibit good ADMET properties in terms of pharmacokinetic parameters.^37^

Protocatechuic acid (3,4-dihydroxybenzoic acid), a powerful antioxidant, creates this effect by reducing lipid peroxidation^38^ and inhibiting DNA fragmentation in hydrogen peroxide (H_2_O_2_)-induced oxidative stress.^39^ Stojković et al. (2013) showed that the antimicrobial activity of protocatechuic acid is due to the significant nucleotide leakage.^40^ In cell culture studies, it was determined that protocatechuic acid reduced ROS-induced apoptosis by preventing lactate dehydrogenase (LDH) release in H_2_O_2_-induced oxidative stress^41^ and by inhibiting intracellular reactive oxygen species ROS levels.^42^ Chlorogenic acids, a secondary metabolite, are esters formed between cinnamic acid derivatives and quinic acid and are important intermediates in lignin biosynthesis in plants. Chlorogenic acid has been determined to have antiphlogistic, antioxidant, antimutagenic, and other biological activities.^43^

### 3.2 Biological Activities of Extracts

The concentration range of 25–400 mg/L was used for the antioxidant assay. Catechin standard was used for the antioxidant assay. It was determined that the DPPH% values of water extract were higher than the values of methanol extracts (Table 2).

It was determined that the difference in DPPH (%) values and the extracts was significant (*P* < .05) except for TFCW. The reduction capacity of plant extracts prepared at different concentrations is related to electron transfer ability and is an important indicator for antioxidant activity results. The absorbance values of the Fe^3+^ ion reduction capacity are given in Table 3. The highest value was determined at *Ep*B. It was observed that the Fe^3+^ reduction capacity increased as the concentration increased in all plant extracts (Table 3). It was determined that there is a two-way, significant positive correlation between TPC and TFC amounts and DPPH (%). Moreira et al. reported that chlorogenic acid contributes to the iron-reducing activity of coffee beverages.^44^ Chlorogenic acid forms complexes with Fe­(III) via hydrogen bonds and is very effective in Fe­(II) chelation and hydroxyl (OH) radical scavenging.^45^

**Table 3:** Optical Density and Reduction Values (%) of Fe^3+^ Reduction Capacity Results in Methanol Extract^a^

	OD ± SD (mg/mL)					reduction values (%)		
extract	100	200	300	400	100	200	300	400
*Ep*LL	0.038 ± 0.003	0.050 ± 0.005	0.077 ± 0.007	0.122 ± 0.009	47.37	50 60	71.43	83.61
*Ep*UL	0.032 ± 0.006	0.055 ± 0.006	0.098 ± 0.007	0.158 ± 0.010	43.75	67.27	81.63	88.61
*Ep*P	0.229 ± 0.006	0.513 ± 0.011	0.698 ± 0.007	0.873 ± 0.014	55.46	80.12	85.39	86.03
*Ep*B	0.051 ± 0.005	0.217 ± 0.007	0.415 ± 0.005	0.552 ± 0.010	33.33	84.33	91.81	93.84
*Ep*F	0.049 ± 0.003	0.062 ± 0.006	0.131 ± 0.006	0.026 ± 0.010	46.94	58.06	80.15	ND
*Ep*R	0.036 ± 0.002	0.051 ± 0.005	0.059 ± 0.004	0.105 ± 0.006	41.66	58.82	64.41	80.00
*Ep*S	0.028 ± 0.004	0.041 ± 0.007	0.053 ± 0.006	0.103 ± 0.004	41.66	58.82	64.41	80.00

a OD: optical density, SD: standard deviation.

In this study, Gram-positive P. aeruginosa ATCC 11778, K. pneumoniae NRLLB 4420, E. coli ATCC 35213, and Gram-negative S. aureus 25292 microorganisms were used. The main differences between Gram-positive and Gram-negative bacteria are based on their cell wall structure. The cell wall structure of bacteria plays a critical role in their susceptibility and resistance to antibiotics. The composition and organization of the cell wall directly affect the mechanisms of action of antibiotics and the resistance mechanisms that bacteria develop to these effects. β-Lactam antibiotics, such as penicillin and cephalosporins, disrupt the integrity of the bacterial cell wall by inhibiting the synthesis of the peptidoglycan layer. These antibiotics inhibit the transpeptidase enzyme involved in cross-linking of peptidoglycan chains, which leads to weakening of the cell wall and lysis of the bacteria. In Gram-negative bacteria, the periplasmic space is located between the outer membrane and the cytoplasmic membrane. This region may contain enzymes that inactivate antibiotics such as β-lactamases. These enzymes break down β-lactam antibiotics, rendering them ineffective and causing the bacteria to become resistant.^46,47^

The effectiveness of a plant extract in treating bacterial diseases depends directly on the type, concentration, and interactions of the bioactive compounds contained in the extract. This activity can be synergistic or antagonistic. Plant extracts are often insufficient to completely eliminate bacterial infections, but they do provide an indirect contribution by supporting the immune system. *Ep*P-M was found to have potent antimicrobial activity against both Gram-positive and Gram-negative bacteria. The highest antimicrobial activity was determined to be against P. aeruginosa (35 ± 0.16). This was followed by K. pneumoniae (23 ± 0.09), E. coli (12 ± 0.05), and S. aureus (11 ± 0.06). Potent antimicrobial activities of E*p*P-M are given in Table 4. HPLC analysis results of E*p*P-M show that the extract contains ferulic acid (15.61 mg/100 g), chlorogenic acid (10.12 mg/100 g), vanillic acid (8.76 mg/100 g), protocatechuic acid (5.78 mg/100 g), and coumaric acid (2.26 mg/100 g). Therefore, these acids were studied in in silico analyses. The PubChem code and chemical structures of ferulic acid, gallic acid, chlorogenic acid, protocatechuic acid, and vanillic acid are given in Table 5.

**Table 4:** Potent Antimicrobial Activity of EpP-M^a^

		zone diameter (mm ± SD)				
gram	microorganism	EpP-M	C30	E(15)	P(10)	DMSO
negative	E. coli (ATCC 35213)	12 ± 0.05	32	23	30	
	K. pneumoniae (NRLLB 4420)	23 ± 0.09	24		6	
	P. aeruginosa (ATCC 11778)	35 ± 0.16	30	13		
positive	S. aureus (ATCC 12600)	11 ± 0.06	19	27	35	

a DMSO: dimethyl sulfoxide, P(10): penicillin, C(30): chloramphenicol, E(15): erythromycin.

**Table 5:**
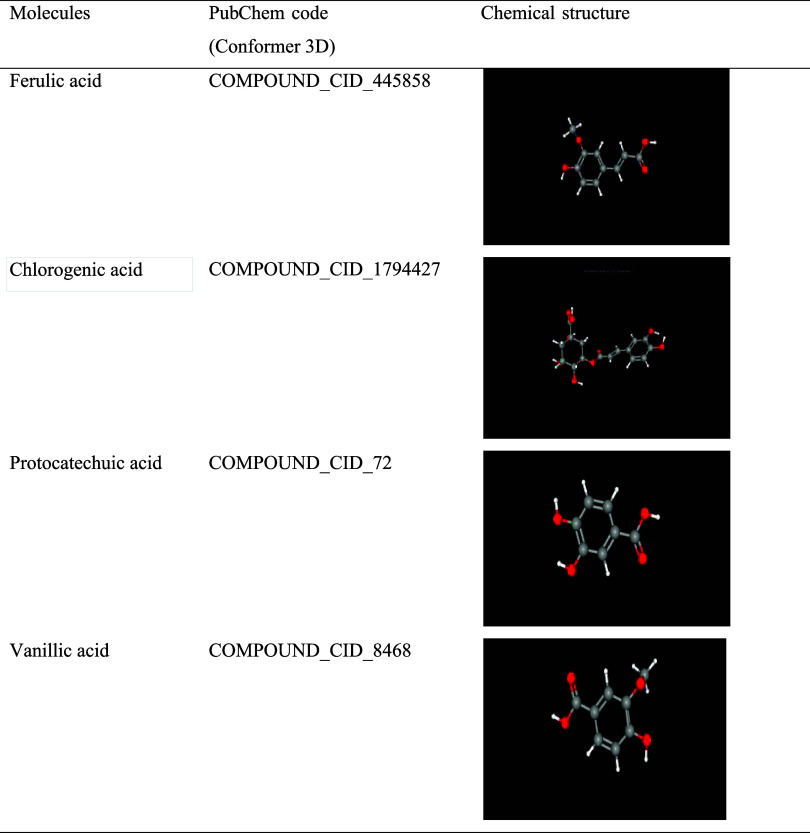
PubChem Code and Chemical Structures of Acids

It has been reported that ferulic acid has antibacterial effects against E. coli O157: H7 ATCC 43888 and Listeria monocytogenes ATCC 7644 ^48^ and S. aureus.^49^ The antibacterial mechanism of ferulic acid is explained by the fact that it causes cell membrane hyperpolarization and, as a result, decreases intracellular pH.^50^ Ergün et al. determined that ferulic acid caused significant changes in membrane properties related to hydrophobicity, and local ruptures or pores were formed in cell membranes.^51^ Vanillic acid (4-hydroxy-3-methoxybenzoic acid) is a metabolite of tyrosine and catecholamine. Vanillic acid has shown its potential as a preservative for Cronobacter sakazakii contamination, but its antibacterial mechanism of action has not yet been exploited. It has been reported that vanillic acid exposure causes a decrease in intracellular pH, ATP, and cell membrane potential in bacterial cells.^52^

Sung et al. showed that chlorogenic acid disrupted the structure of fungal cell membranes; similarly, Lou et al. showed that chlorogenic acid disrupted membrane permeability by irreversibly changing the cell membrane potential.^53,54^ Chlorogenic acid can also be considered as a potential inhibitor of efflux pumps and biofilm formation and may offer potential strategies to overcome antimicrobial resistance.^55^

To assess ligand binding affinities accurately, molecular docking coring proved inadequate, prompting the use of MM/PB­(GB)­SA analyses. The MM/PB­(GB)­SA graph illustrating ligand affinities is depicted in Figure 3.

**Figure 3:**
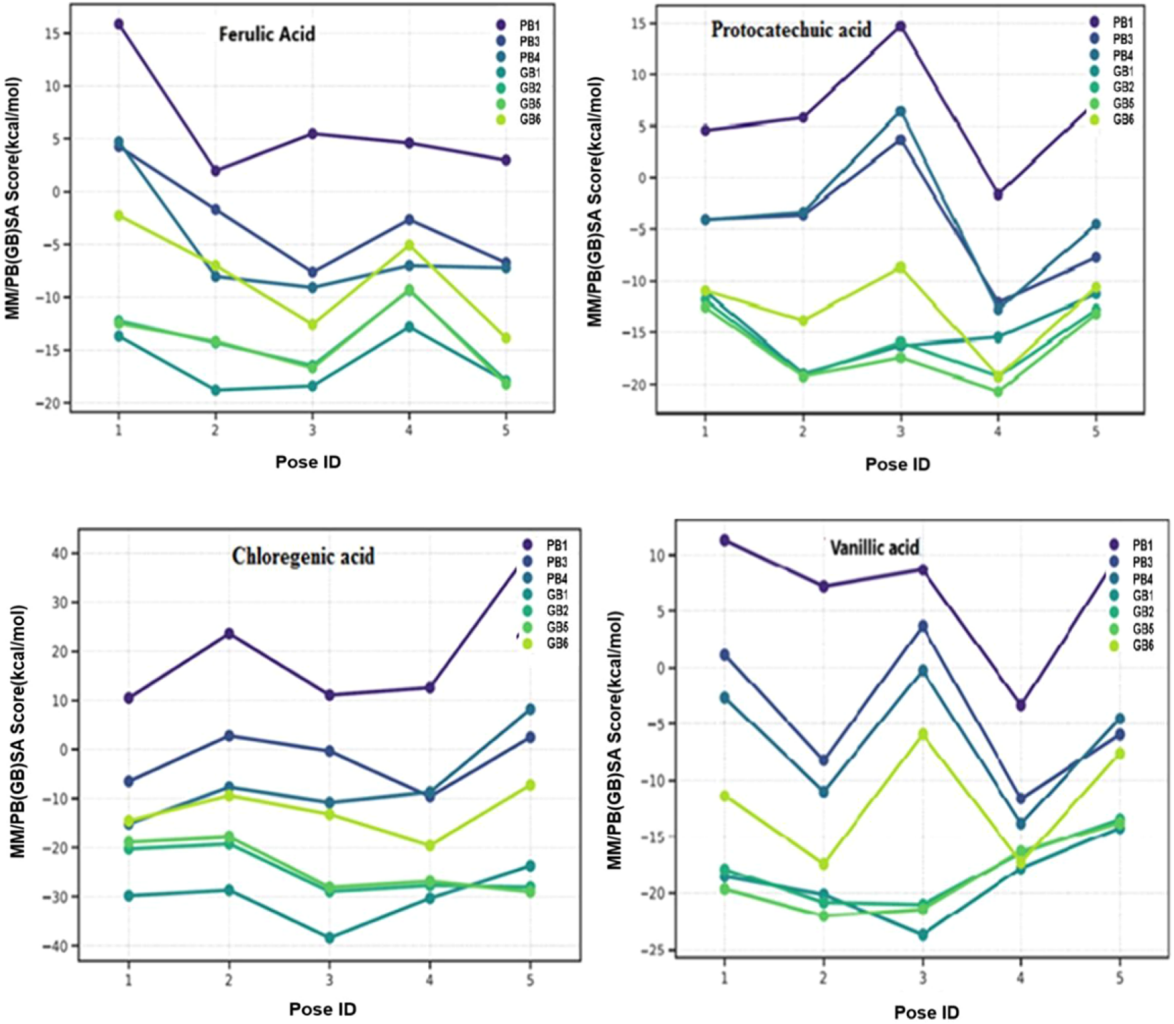
Binding free energy (MM/PB­(GB)) SA graph of acids.

The lowest protein–ligand binding energy (kcal/mol) was found to be −9.6 kcal/mol in chlorogenic acid. Chlorogenic acid can bind with PPAT’s T10­(B)­OG1, W12­(b), I127­(B)­O, and S129­(B)­OG and P88­(B)­HN2, T10­(B)­N, F11­(b)­N, D12­(B)­N, S129­(B)­N, and K12­(B)­NZ receptors via hydrogen bonds. Negative binding energy (kcal/mol) values indicate that the reaction is exothermic and occurs voluntarily. PPAT protein–ligand binding energy (kcal/mol) was found to be −6.9 kcal/mol for ferulic acid, −6.5 kcal/mol for protocatechuic acid, and −6.5 kcal/mol for vanillic acid (Table 6).^56^

Using WebGro, a molecular dynamics simulation was conducted to evaluate the stability of the docked complexes between ligands and the PPAT protein.

Docked complexes (PPAT–ferulic acid, PPAT–chlorogenic acid, PPAT–protocatechuic acid, and PPAT–vanillic acid) were subjected to molecular dynamics simulations. Molecular docking analyses between the PPAT protein and acids and the hydrogen bond location are given in Table 6. The RMSD technique was employed to assess the conformational stability of the protein backbone and the ligand–protein complexes. Molecular dynamics results of PPAT and chlorogenic acid–PPAT interaction are given in Figure 4.

**Table 6:**
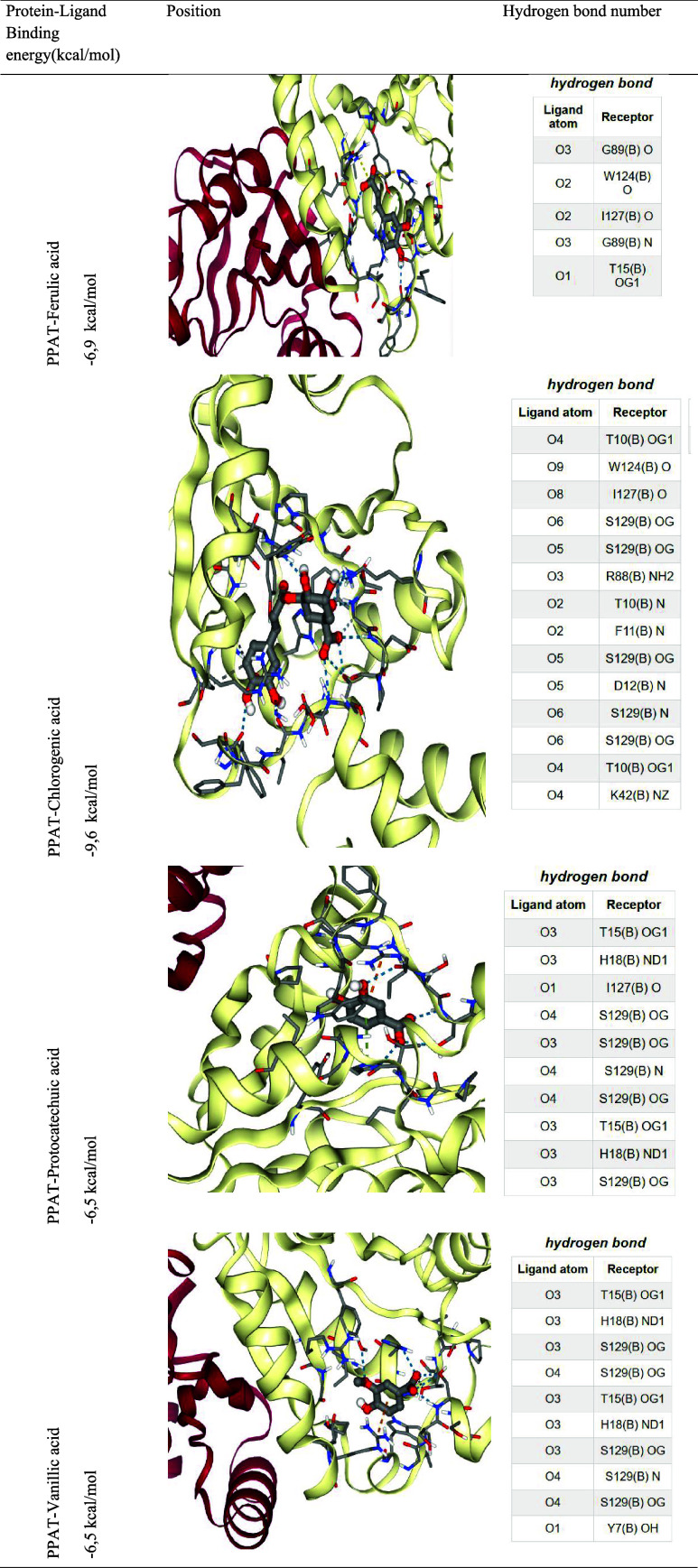
Protein–Ligand Binding Energy and Hydrogen Bonds

**Figure 4:**
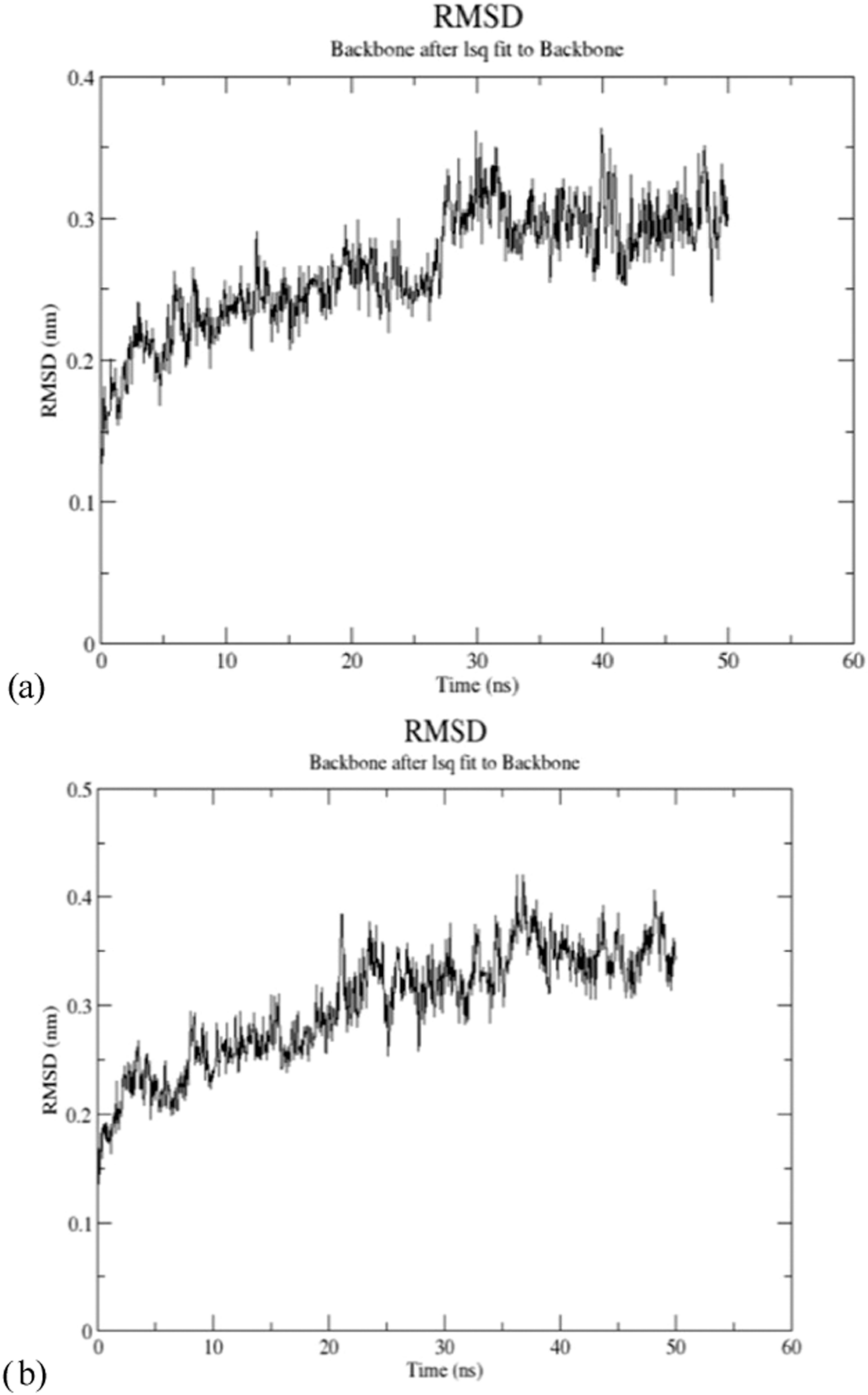
Molecular dynamics results: (a) PPAT and (b) chlorogenic acid–PPAT.

PPAT, which is the product of the *Coa*D gene, plays a crucial role in the synthesis of coenzyme A (CoA), a vital cofactor facilitating various key biochemical processes such as the TCA cycle and fatty acid metabolism.^5,57–59^ It exists in a hexameric form and functions by catalyzing the reversible transfer of the adenylyl group from ATP to 4′-phosphopantetheine, resulting in the production of dephospho-CoA and inorganic pyrophosphate.^60,61^ If this enzyme is inhibited by using chemicals that target the enzyme, microbial CoA biosynthesis can be prevented. The development of selective inhibitors of bacterial PPAT is promising for the discovery of new antibiotics.^6^ In fact, some studies have been conducted to develop new antibacterial agents by targeting PPAT, and various types of inhibitors have been developed.^62^ One of them is dimethoxypyrimidine, which is responsible for antimicrobial activity against both Gram-positive and Gram-negative bacteria.^63^

As a result of this, the inhibition of PPAT by chlorogenic acid validates for the first time PPAT as a novel target for antibacterial therapy.

Ferulic acid, chlorogenic acid, protocatechuic acid, and vanillic acid ligands were tested for ADME by the SwissADME web server to compile the information on the pharmacokinetics and pharmacodynamics of candidate molecules. The drug-likeness was provided by ligands according to the SwissADME predictions (Table 7).

**Table 7:**
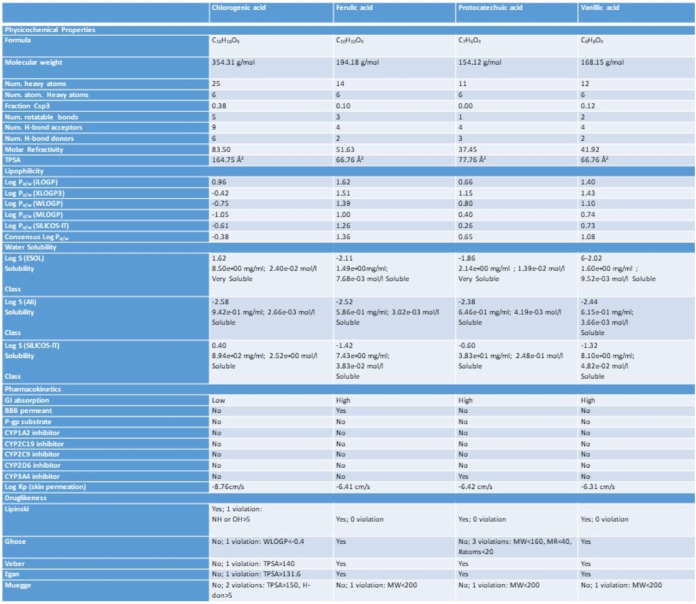
Pharmacokinetics and Drug-Likeness Analyses

Since PPAT is responsible for the synthesis of CoA in bacteria, herbal extracts that inhibit this enzyme disrupt the energy metabolism and fatty acid synthesis of bacteria. As a result, bacterial growth stops, and cellular functions collapse. Since CoA biosynthesis in humans proceeds through different pathways, drugs targeting bacterial PPAT can only affect bacteria without harming human cells. This provides selective toxicity. Bacteria resistant to traditional antibiotics may be sensitive to new drugs targeting PPAT, and therefore, these herbal extracts may be promising against MDR bacteria.

## 4 Conclusions

The development of bacterial resistance to antibiotics is an increasingly important problem in the treatment of infectious diseases. The structural classes of antibiotics we use today are similar to the structural classes discovered in the middle of the last century. Therefore, the development of antimicrobial compounds that target new cellular structures, such as enzymes, has become mandatory today. We detected that E. purpurea extracts had strong antioxidant and antibacterial effects. Especially *Ep*P-M, containing high amounts of ferulic acid (15.61 mg/100 g) and chlorogenic acid (10.12 mg/100 g), shows antimicrobial activity against both Gram-positive and Gram-negative bacteria. Lou et al. hypothesized that the strong antimicrobial activity of chlorogenic acid might be due to its ability not only to permeabilize the membrane but also to target intracellular processes in bacteria. According to our results, the inhibition of PPAT protein by chlorogenic acid validates for the first time PPAT as a novel target for antibacterial therapy.

## Data Availability

The data underlying this article will be shared on reasonable request to the corresponding author.

## Notes

Ethical Statement: This study did not include any humans or animals; therefore, we did not apply for ethical approval or register for clinical trials.
